# Is the Matrix Completion of Reduced Density Matrices Unique?

**DOI:** 10.1021/acs.jpclett.6c00296

**Published:** 2026-03-13

**Authors:** Gustavo E. Massaccesi, Ofelia B. Oña, Luis Lain, Alicia Torre, Juan E. Peralta, Diego R. Alcoba, Gustavo E. Scuseria

**Affiliations:** † Departamento de Ciencias Exactas, Ciclo Básico Común, Universidad de Buenos Aire, Ciudad Universitaria, 1428 Buenos Aires, Argentina and Instituto de Investigaciones Matemáticas “Luis A. Santaló” (IMAS), Consejo Nacional de Investigaciones Científicas y Técnicas, Universidad de Buenos Aires. Ciudad Universitaria, 1428 Buenos Aires, Argentina; ‡ Instituto de Investigaciones Fisicoquímicas Teóricas y Aplicadas, Universidad Nacional de La Plata, Consejo Nacional de Investigaciones Científicas y Técnicas. Diag.123 y 64 (S/N), Sucursal 4, CC 16, 1900 La Plata, Argentina; ¶ Department of Physical Chemistry, Faculty of Science and Technology, University of the Basque Country. PO Box 644, E-48080 Bilbao, Spain; § Department of Physics, 5649 Central Michigan University, Mount Pleasant, Michigan 48859, United States; ∥ Universidad de Buenos Aires, Facultad de Ciencias Exactas y Naturales, Departamento de Física. Ciudad Universitaria, 1428 Buenos Aires, 62873 Argentina and CONICET - Universidad de Buenos Aires, Instituto de Física de Buenos Aires (IFIBA). Ciudad Universitaria, 1428 Buenos Aires, Argentina; ⊥ Department of Chemistry, 3990 Rice University, Houston, Texas 77005-1892, United States; # Department of Physics and Astronomy, 3990 Rice University, Houston, Texas 77005-1892, United States

## Abstract

Reduced density matrices are central to describing observables in many-body quantum systems. In electronic structure theory, the two-particle reduced density matrix (2-RDM) suffices to determine the energy and other key properties. Recent work has used matrix completion, leveraging the low-rank structure of RDMs and approximate theoretical models, to reconstruct the 2-RDM from partial data and thus reduce the computational cost. However, matrix completion is, in general, an under-determined problem. Revisiting Rosina’s theorem ( RosinaM., Queen’s Papers on Pure and Applied Mathematics, 1968, No. 11, 369 ), we here show that the matrix completion is unique under certain conditions, identifying the subset of 2-RDM elements that enables its exact reconstruction from incomplete information. Building on this, we introduce a hybrid quantum–stochastic algorithm that achieves exact matrix completion, demonstrated through applications to the Fermi–Hubbard model.

## Graphical abstract

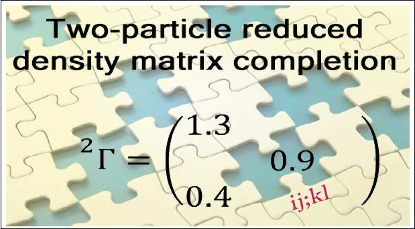

The two-particle reduced density matrix (2-RDM) corresponding to an *N*-particle wave function Ψ is defined by^1,2^

1 Γ2(x1,x2;x1′,x2′)=∫ΓN(x1,x2,x3,...,xN;x1′,x2′,x3,...,xN)dx3...dxN

where ^*N*^Γ­(**x**; **x′**) = Ψ­(**x**)­Ψ*­(**x′**) is the *N*-particle density matrix, and normalization has been omitted for clarity. This matrix carries all of the relevant information needed to evaluate expectation values of one- and two-particle operators, as is often the case. For example, the energy for a pairwise interacting *N*-particle system may be exactly written as^3^

2 EΨ=∑ij;klHij;kl2Γij;kl2

where {^2^H_*ij*;*kl*_} is the two-particle reduced Hamiltonian

3 Γij;kl2≡∫φi*(x1)φj*(x2)Γ2(x1,x2;x1′,x2′)φk(x1′)φl(x2′)dx1dx2dx1′dx2′=⟨Ψ|âi†âj†âlâk|Ψ⟩

and ${â}_{i}^{†}$ and ${â}_{i}$ denote particle creation and annihilation operators acting on a finite-size single-particle basis {φ_*i*_}, respectively. The 2-RDM is a much more compact and economic storage of information than the *N*-particle wave function.^1,4^ However, while the wave function Ψ must satisfy appropriate exchange symmetry and normalization, the 2-RDM must satisfy the so-called *N*-representability conditions,^3,5–8^ which bear a complexity that grows exponentially with *N*, in order to fulfill eq 1.^8–10^

Equation 1 provides a simple prescription to calculate the 2-RDM given a preimage Ψ. The inverse problem, i.e., given a 2-RDM, how to derive a preimage Ψ, is known as the reconstruction problem. This problem lies at the heart of quantum state and process tomography, which are fundamental techniques used to characterize unknown quantum states and processes as well as to quantify the quality of quantum devices.^9,11,12^ As it will be shown in this Letter, the reconstruction problem is intimately related to the completion problem, which refers to the process of completing the full, physically valid 2-RDM from a partial subset of its elements (typically those that are directly measured or approximated).

In 1968, Rosina proved a theorem that establishes conditions such that the reconstruction is unique.^13^ The theorem shows that the 2-RDM corresponding to a nondegenerate ground state of a quantum system completely determines the exact *N*-particle wave function without any specific information about the Hamiltonian other than bearing at most two-particle interactions.^14,15^ In this Letter we demonstrate that the subset of elements of the 2-RDM needed for a unique reconstruction of the preimage Ψ, and hence for the matrix completion of the full 2-RDM, is linked to the subset of nonzero elements of the two-particle reduced Hamiltonian {^2^H_*ij*;*kl*_}.

**Theorem 1**. (“*Uniqueness RDM Completion Theorem*”) *For an *N*-particle Hamiltonian with at most two-particle interactions with reduced representation* {^2^H_*ij*;*kl*_} *and a nondegenerate ground state, the subset of elements of the 2-RDM corresponding to the ground state associated with the nonzero elements of* {^2^H_*ij*;*kl*_} *has a unique preimage, leading to a unique matrix completion of the full 2-RDM via* eq 1.

*Proof*. Let *S* be the subset of indices for which the elements of the two-particle reduced Hamiltonian {^2^H_*ij*;*kl*_} are nonzero. The energy, eq 2, may be calculated as follows:

4 EΨ=∑(ij;kl)∈SHij;kl2Γij;kl2

Since the Hamiltonian consists solely of two-particle operators, the ground-state energy is completely determined by the 2-RDM elements in *S* and is minimized by the exact ground-state solution. If there existed another *N*-particle-density matrix ^*N*^Γ yielding the same 2-RDM elements in *S* via eq 1, it would necessarily produce the same energy and thus also correspond to a ground state (or an ensemble of ground states), as only ground states attain minimal energy. This would contradict the assumed nondegeneracy of the ground state. Finally, like in Rosina’s theorem,^13–17^ since a nondegenerate ground state cannot be represented as a nontrivial ensemble, such a subset of elements of the 2-RDM must admit a pure-state preimage. In consequence, this leads to a *unique* matrix completion of the 2-RDM via eq 1.

We note that only the location of the subset *S* in the 2-particle reduced Hamiltonian is needed for completion, not the actual matrix element values. Moreover, subset *S* is basis-dependent, and thus, the number of matrix elements needed for the completion varies with the representation. To illustrate how the Theorem manifests in practical applications, we utilize a hybrid quantum–stochastic algorithm that numerically performs the matrix completion of a 2-RDM. The algorithm consists of applying to an initial *N*-particle density matrix a sequence of unitary evolution operators generated by a stochastic process that iteratively refines the partial information encoded in the reduced two-particle state. A similar algorithm has been employed by us to numerically determine the *N*-representability of a pure RDM.^18^ Through this iterative procedure, the elements of the 2-RDM converge toward the critical subset of elements of a target 2-RDM associated with the nondegenerate ground state of an *N*-particle Hamiltonian involving at most two-particle interactions, thereby enabling its exact completion. A summary of the procedure is shown in Algorithm [Fig sch1].^19^

**Scheme 1:**
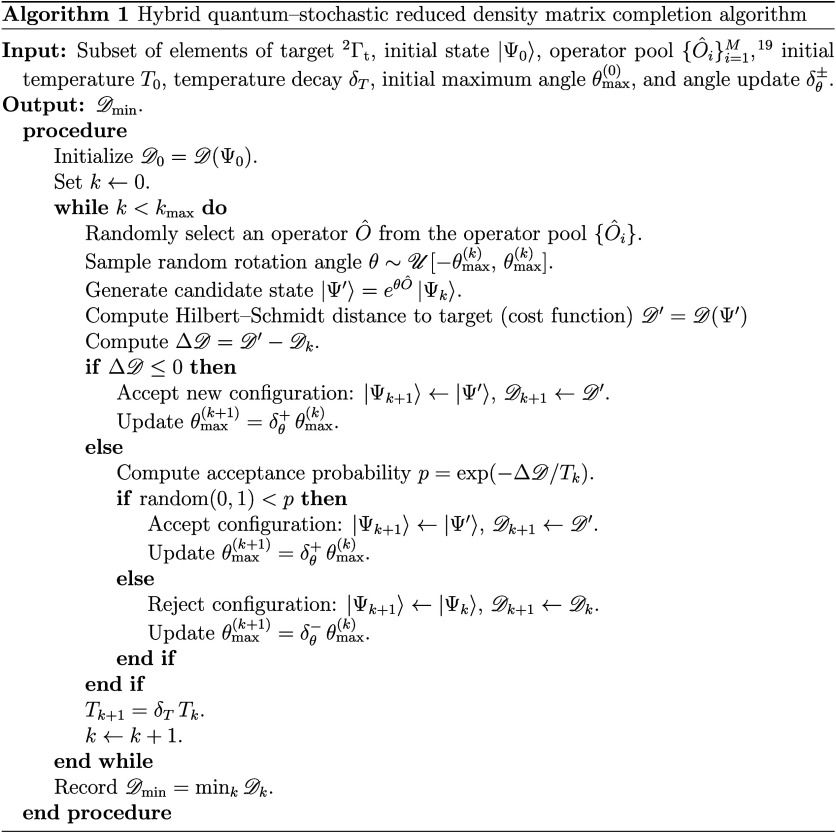


As a proof-of-concept, we consider the ground state of the inhomogeneous Fermi–Hubbard model for a three-site one-dimensional lattice with open boundary conditions at half-filling, defined by the Hamiltonian^20^

5 $$Ĥ=-\underset{\left\langle i,j\right\rangle ,\sigma }{\sum }\left({â}_{i\sigma }^{†}{â}_{j\sigma }+{â}_{j\sigma }^{†}{â}_{i\sigma }\right)+\underset{i,\sigma }{\sum }\epsilon_{i\sigma }{\mathrm{n̂}}_{i\sigma }+U\underset{i}{\sum }{\mathrm{n̂}}_{i↑}{\mathrm{n̂}}_{i↓}$$

where ${â}_{i\sigma }^{†}\left({â}_{i\sigma }\right)$ denotes the Fermionic creation (annihilation) operator for a particle at site *i* with spin σ (*↑*, *↓*), and ${\mathrm{n̂}}_{i\sigma }={â}_{i\sigma }^{†}{â}_{i\sigma }$ is the corresponding number operator. The notation ⟨*i*, *j*⟩ indicates nearest-neighbor pairs on the lattice. The on-site energy parameters ϵ_*i*_ are introduced to break spin and spatial symmetries and remove ground-state degeneracies, and the on-site interaction strength *U* is assumed to be positive.

In the lattice-site basis, the target 2-RDM corresponding to the nondegenerate ground state comprises 900 elements, out of which 360 fulfill *S*_*z*_-spin symmetry and are nonzero. Among these, 184 elements correspond to the symmetrized nonzero elements of the two-particle reduced Hamiltonian, while the remaining elements are determined under the conditions established in Theorem 1, as illustrated in Figure 1. The left panel of Figure 3 shows the evolution of the partial (critical subset of elements) and complete (full set of elements) Hilbert–Schmidt distances between the reduced two-particle state of the unitarily evolved *N*-particle density matrix and the target 2-RDM, respectively, obtained from the completion algorithm. The corresponding energy deviation and infidelity of the evolved *N*-particle density matrix from the exact ground state are also presented in the right panel of Figure 3. The evolution is initiated from an arbitrary linear combination of the ground and first excited states. Overall, the numerical results shown in Figures 1 and 3 demonstrate that the elements of the evolved 2-RDM progressively converge toward both the critical subset and the remaining components of the target 2-RDM associated with the nondegenerate ground state, thereby demonstrating exact completion. An analogous behavior is observed in the eigenbasis of the two-particle reduced Hamiltonian. In this representation, 27 elements of the target 2-RDM correspond to the symmetrized nonzero reduced two-particle Hamiltonian entries, while the remaining elements are determined by the conditions stated in Theorem 1, as illustrated in Figure 2. As in the lattice case, evolution from a generic low-energy superposition leads to convergence to the exact ground-state 2-RDM, confirming the robustness and generality of the matrix completion scheme across different representations. Choosing an eigenbasis representation may be particularly useful in systems in which symmetry cannot be exploited, such as asymmetric molecules.

**Figure 1:**
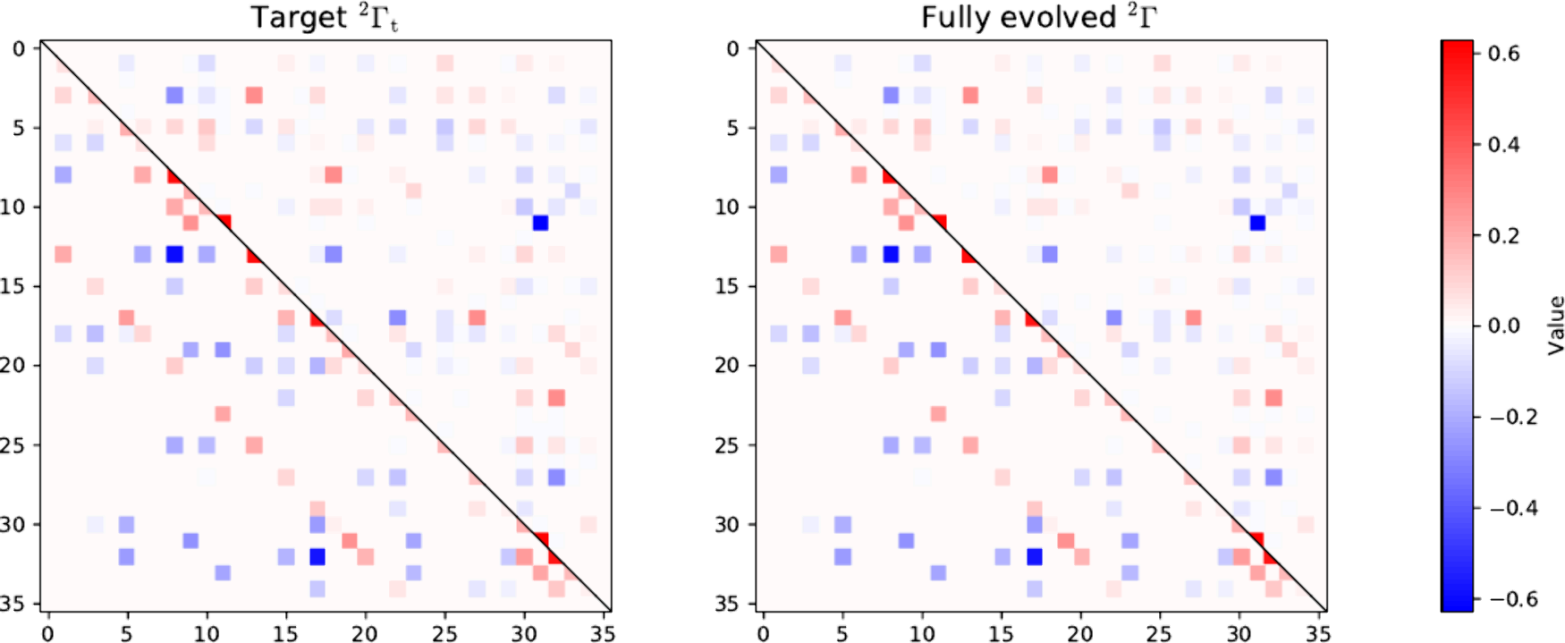
Heat-map representations in the lattice-site basis of the target 2-RDM (left panel), and the reconstructed (fully evolved) density-matrix elements obtained via matrix completion (right panel) corresponding to the nondegenerate ground state of the inhomogeneous three-site one-dimensional Fermi–Hubbard model with open boundary conditions at half-filling. Density-matrix elements associated with the symmetrized nonzero (zero) elements of the corresponding two-particle reduced Hamiltonian are shown as the lower (upper) triangular part of the matrices.

**Figure 2:**
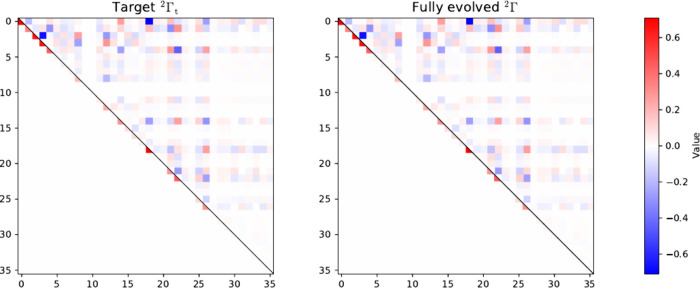
Heat-map representations in the eigenbasis of the two-particle reduced Hamiltonian of the target 2-RDM (left panel), and the reconstructed (fully evolved) density-matrix elements obtained via matrix completion (right panel) corresponding to the nondegenerate ground state of the inhomogeneous three-site one-dimensional Fermi–Hubbard model with open boundary conditions at half-filling. Density-matrix elements associated with the symmetrized nonzero (zero) elements of the corresponding two-particle reduced Hamiltonian are shown as the lower (upper) triangular part of the matrices.

**Figure 3:**
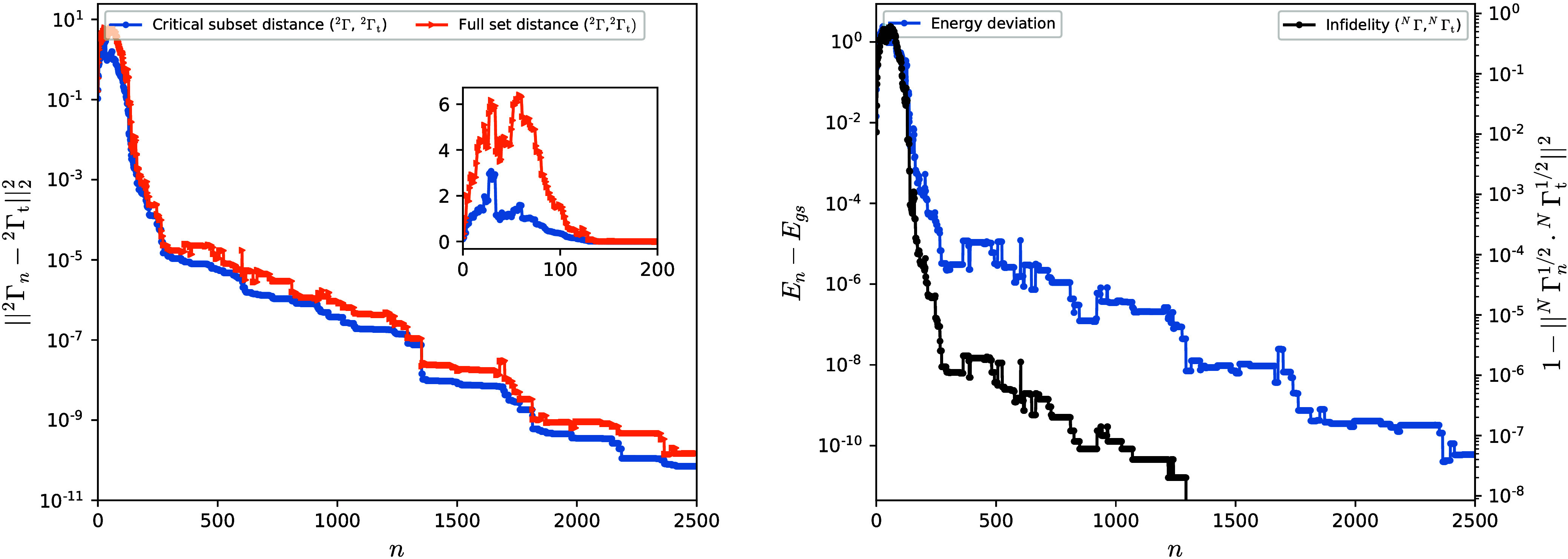
Left panel: Partial (critical subset of elements) and complete (full set of elements) Hilbert–Schmidt distances between the reduced two-particle state of the unitarily evolved *N*-particle density matrix, ^2^Γ, and the target 2-RDM, ^2^Γ_t_, respectively, as a function of iteration number *n*, for the exact ground state of the model system in Figure 1. The evolution starts from an arbitrary linear combination of the ground and first-excited states. Inset: enlarged view of the first 200 iterations. Right panel: Energy deviation and infidelity of the evolved *N*-particle density matrix with respect to the exact ground-state density matrix as a function of iteration number *n*.

Thus far, we have evaluated our algorithm in the noiseless matrix completion setting, where the partial information consists of the critical subset of 2-RDM elements associated with a nondegenerate ground state. To assess the robustness of the methodology under more challenging conditions, we introduce statistical noise into the target 2-RDM on the lattice-site basis, ^2^Γ_t_, while retaining the same subset of known elements. Specifically, we define

6 Γt2(ε)=Γt2+εR

where *R* has elements drawn from a uniform distribution in [−1, 1] and the parameter *ε* ∈ [0, 0.1] controls the noise strength. The chosen values of *ε* produce noticeable distortions in the target 2-RDM. In this regime, the algorithm is expected to converge toward the target only up to a noise-dependent limit: larger noise strengths should yield larger $D_{\min}$ , while in the noiseless case $D_{\min}\to 0$ , as previously demonstrated. Table 1 confirms this expectation, showing that the converged Hilbert–Schmidt distances for ^2^Γ_t_(*ε*) increase as the noise strength increases. These results emphasize applications of the proposed algorithm: It can be used not only in noiseless matrix completion settings but also in noisy ones, constructing a 2-RDM (the evolved RDM) that is closest to the target.

**Table 1:** Partial (Critical Subset of Elements) and Complete (Full Set of Elements) Hilbert–Schmidt Distances between the Reduced Two-Particle State of the Unitarily Evolved *N*-Particle Density Matrix, ^2^Γ, and the Noiseless and Noisy Target 2-RDMs, ^2^Γ_t_ and ^2^Γ_t_(*ε*), Respectively, for the Exact Ground State of the Model System in Figure 1^a^

	Hilbert–Schmidt distance		
	Critical subset		Full set
ε	(^2^Γ, ^2^Γ_t_(*ε*))	(^2^Γ, ^2^Γ_t_)	(^2^Γ, ^2^Γ_t_)
10^–3^	2.99 × 10^–5^	2.44 × 10^–5^	1.28 × 10^–4^
10^–2^	3.22 × 10^–3^	2.44 × 10^–3^	1.52 × 10^–2^
10^–1^	5.94 × 10^–1^	2.52 × 10^–1^	4.08 × 10^–1^

a The noisy targets are constructed by adding random noise of strength *ε* to the noiseless 2-RDM (see text for details).

Rosina’s theorem proves that the 2-RDM corresponding to a nondegenerate ground state of a quantum system completely determines the exact *N*-particle wave function without any specific information about the Hamiltonian other than bearing at most two-particle interactions. Building on that theorem, we derive the proof that rigorous matrix completion uniqueness conditions exist. The subset of elements of the 2-RDM needed for the determination of the unique preimage Ψ, and hence for a full reconstruction of the 2-RDM, is linked to the subset of nonzero elements of the two-particle reduced Hamiltonian. This result establishes clear conditions under which the 2-RDM can be uniquely reconstructed from incomplete information, thereby strengthening the theoretical underpinnings of matrix completion in the electronic structure theory. Moreover, the formal conditions established here support future developments in quantum tomography^21^ by identifying the minimal information needed to reconstruct physically valid two-particle correlations. The framework also provides a systematic way to correct defective or noisy RDMs, making it relevant for error mitigation techniques^22^ on near term quantum devices,^23,24^ and complements recent efforts to develop matrix completion strategies for Fermionic RDMs.^25^ As a numerical proof-of-concept, we have here demonstrated its applicability using a hybrid quantum–stochastic algorithm that achieves unique matrix completion of the full 2-RDM of a Fermi–Hubbard model from a subset of it.
